# The Book World of Medicine and Science

**Published:** 1900-02-10

**Authors:** 


### The Book World of Medicine and Science.

Letter, Word, and Mind Blindness. By James Hinshelwood, M.A., M.D., Surgeon to the Glasgow Eye Infirmary. (H. K. Lewis. Pp. 83. Price 3s.)

This book consists of five chapters, four of which have already appeared in the *Lancet*, and it is certainly desirable they should have a circulation in permanent form. Dr. Hinshelwood begins by saying that while the anatomy, physiology, pathology, and physics of the eye have been investigated with the greatest care, the cerebral or mental aspect has not received the same attention. No doubt this is so, but we think these chapters constitute a considerable stride in the direction of making up the existing deficiency. The chapter on visual memory, not hitherto published by Dr. Hinshelwood, shows us what an important part the education of this visual memory plays in the production of the complex phenomena which we group under the word "sight," and also how little the, so to speak, mere mechanical part of vision could be relied on until this mental storage of facts and impressions has been obtained. Everyone possesses the visual memory to a greater or lesser extent, and occasionally it is developed to an extraordinary degree. Dr. Hinshelwood mentions the case of the French artist Vernet, and Wigan tells us he knew a portrait-painter who never required more than one sitting from his subject. After that he could recall the figure, face, and attitude of the sitter, and thus finish the picture. In this case it would hardly be going too far to say that the artist actually saw the individual whose portrait he was painting, and although the man subsequently went insane, it might be regarded as an extreme case of healthy development, the stimulus coming from within, the image voluntarily called up and the "seer" perfectly recognising that his subject was not actually in the chair before him. When involuntary, and not corrected by a normal process of reasoning, we have an explanation of one form of the perception of ghosts and spirits exemplifying an unhealthy visual memory. Just as this visual memory may be abnormally developed so it may be entirely lost. Dr. Hinshelwood gives us details of a patient of his in whom this happened, and the man had in adult age to begin learning to read printed matter again, but oddly enough his memory for figures was not impaired. Not only word blindness but musical note blindness is known to exist, and Edgren, of Stockholm, has collected fifty-two cases of this disease, which he names "amusia." The concluding chapters are on "partial mind blindness" and "letter-without-word blindness." This work may without reserve be recommended, as it contains much information given in clear and sensible language. It comes to us unweighted with irrelevant matter.

